# Trichorhinophalangeal Syndrome Type I: A Patient with Two Novel and Different Mutations in the TRPS1 Gene

**DOI:** 10.1155/2013/748057

**Published:** 2013-04-17

**Authors:** Catarina Dias, Lara Isidoro, Mafalda Santos, Helena Santos, Jorge Sales Marques

**Affiliations:** ^1^Paediatrics Department, Centro Hospitalar de Vila Nova de Gaia/Espinho, EPE, Unidade II, 4400-129 Vila Nova de Gaia, Portugal; ^2^Orthopaedics Department, Centro Hospitalar de Vila Nova de Gaia/Espinho, EPE, Unidade II, 4400-129 Vila Nova de Gaia, Portugal

## Abstract

*Background*. Trichorhinophalangeal syndrome (TRPS) is an autosomal dominant skeletal dysplasia caused by defects involving the TRPS1 gene. Three types (TRPSs I, II, and III) have been described, exhibiting the common triad of hair, craniofacial, and skeletal abnormalities. TRPS II includes the additional characteristics of mental retardation and multiple exostoses. *Case Report*. We describe a sporadic case of TRPS type I in a child with two novel nonsense pathogenic mutations in the TRPS1 gene, both in heterozygosity—c.1198C>T (p. Gln400X) and c.2086C>T (p.Arg696X). None of these mutations were found in her parents. Clinical presentation included typical hair and facial features, as well as slight skeletal abnormalities. *Discussion*. There is a wide variability in clinical expression of TRPS I. Manifestations of the disease can be subtle, yet skeletal anomalies imply that TRPS I is more than an esthetic problem. Clinical and genetic diagnosis allows adequate followup and timely therapeutic procedures. When a single mutation was sufficient for the onset of the disease, our patient presented two different ones.

## 1. Introduction

Trichorhinophalangeal syndrome (TRPS) is a rare skeletal dysplasia of autosomal dominant inheritance, caused by a defect in the TRPS1 gene. This gene, located on chromosome 8 (8q24.1) [1], encodes a transcriptional repressor involved in hair development and chondrocyte modulation [2].

Key clinical features of the syndrome include fine, slowly growing hair, a high frontal hairline, and rarefaction of the lateral eyebrows; craniofacial peculiarities with a typical pear-shaped nose, long and flat philtrum, thin upper lip, receding chin, and protruding ears; phalangeal cone-shaped epiphyses—resulting in brachydactyly or clinodactyly—and other orthopedic abnormalities, such as hip malformation and short stature [3–6]. 

Three types of trichorhinophalangeal syndrome have been described: TRPS I (Online Mendelian Inheritance in Man [OMIM] 190350), TRPS II (OMIM 150230), and TRPS III (OMIM 190351) [7, 8]. TRPS I, first described by Giedion, in 1966 [3], may be caused by several deletions or mutations in the TRPS1 gene [4, 7]. TRPS II, or Langer-Giedion syndrome, is a contiguous gene syndrome due to loss of functional copies of both the TRPS1 and EXT1 genes. It differs from TRPS I by the presence of mental retardation and multiple cartilaginous exostoses, in addition to the TRPS phenotype [9]. TRPS III has similar clinical features to TRPS I but presents with severe brachydactyly (due to short metacarpals) and growth retardation [4].

So far, a few hundred cases of TRPS have been described in the literature, involving dozens of different mutations [4, 10–16]. There are probably some more who remain undiagnosed, as clinical features can be mild. We present the case of a child with two novel and different mutations, not previously reported.

## 2. Case Presentation

A 4-year-old girl was observed in the emergency department after minor nose trauma. Clinical examination was negative for traumatic injury but disclosed a bulbous nose, as well as thin and short scalp hair, which had never been trimmed. She was referred to a pediatrics consultation for further dysmorphism investigation.

She was born from young, healthy, and nonconsanguineous parents and had an unremarkable personal history, except for chronic nasal obstruction and snoring. 

**Figure 1:**
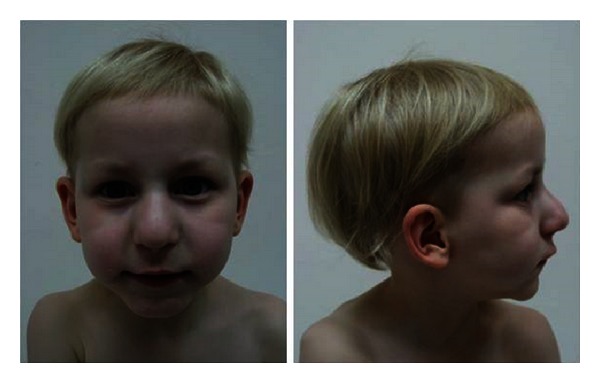
A 4-year-old girl presenting typical craniofacial TRPS features: fine hair, a bulbous nose, long philtrum, thin lips, and a receding chin.

She presented adequate growth (weight in 5th, height in 10th, and head circumference in 75th percentiles) and psychomotor development. Her physical examination showed a pear-shaped nose, as well as a long philtrum, thin lips, a broad forehead, and rarefaction of the lateral eyebrows. She presented a lighter complexion than her family, with blue eyes and blonde hair (Figure 1). Her nails had a soft consistency; the 5th toenails were reported to have a very slow growth rate and had never required a cut. Bilateral inward curving of the 2nd toes was the only orthopedic abnormality found. She had a hyperkinetic behavior during observation. 

**Figure 2:**
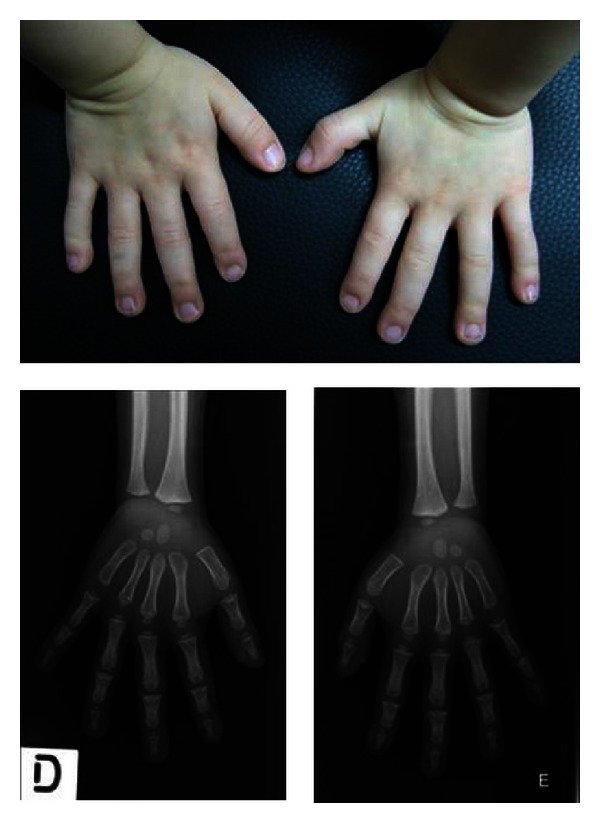
Photograph and radiograph of a 4-year-old girl's hands, showing short metacarpals and metaphyseal concavity involving the proximal phalanges of the thumb and small finger and all the middle phalanges.

Radiographic studies revealed abnormal proximal and middle phalanges of toes and fingers (Figure 2). Bone age was two and a half years behind her chronological age.

Neither her parents nor her older brother manifested the disease phenotype. Given the clinical characteristics, the diagnosis of a sporadic case of TRPS type 1 was then considered very likely. 

Genetic analysis was performed through gene sequencing of the 5 coding exons and their flanking intronic sequences in the TRPS1 gene. Two novel nonsense pathogenic mutations were identified in exon 4, both in heterozygosity—c.1198C>T (p.Gln400X) and c.2086C>T (p.Arg696X) (the authors can provide additional data related to the primer sets and PCR conditions for DNA sequencing analyses of the TRPS1 mutations they determined in this study). None of these mutations were found in her parents.

The patient was referred to an otorhinolaryngology consultation and an adenoamigdalectomy was performed, with clinical benefit. No hearing deficit was identified. 

She was also evaluated in an orthopedic consultation, where other types of skeletal malformations, namely, on the hip, were excluded. Presently, at 6 years of age, her osteoarticular system is only lightly affected, showing short metacarpals and a slight leg length inequality (of 1 cm), as well as bilateral inward curving of the 2nd toes. 

Her hyperkinetic behavior became more noticeable as she entered elementary school. An attention deficit/hyperactivity disorder (ADHD) was diagnosed and she was given methylphenidate, with immediate clinical improvement. Formal cognitive evaluation assessed by the Griffiths Mental Development Scales-Extended Revised: 2 to 8 years (GMDS-ER 2–8) revealed normal development for her age. 

## 3. Discussion

### 3.1. TRPS1 Gene Defects and Clinical Manifestations

TRPS1, identified and mapped in 2000 by Momeni et al. [1], encodes a 141-kd protein composed of 1281 amino acids, with at least one nuclear localization signal and a combination of different zinc-finger motifs, including IKAROS-like and GATA-like binding sequences. It functions as a transcription factor that represses GATA-regulated genes [8]. 

It has been shown that TRPS type I is associated with deletions, nonsense, and missense mutations of one allele of TRPS1 [4, 7]. The mutant, truncated protein (lacking nuclear localization signals) cannot enter the nucleus and exert its function—a phenomenon known as haploinsufficiency [13]. In contrast, the more severe TRPS III phenotype has been found to be exclusively associated with missense mutations in the GATA zinc finger of TRPS1; the resulting abnormal protein possibly competes with the wild-type TRPS1 in a multimeric transcription control complex, exerting a dominant-negative effect [4]—that explains why a complete deletion of the GATA zinc finger has milder consequences than a missense mutation in it.

At the present date, more than 50 mutations have been found [4, 10–16], the majority of them being responsible for sporadic cases. To the best of our knowledge, our patient presents with two novel mutations in the TRPS1 gene. Both of them are nonsense mutations, potentially resulting in a premature stop codon and an abnormally truncated TRPS1 protein, which lacks the C-terminal region, including the GATA zinc finger. The molecular study performed could not differentiate whether the two sequence changes are in *cis* (coexisting in the same allele) or *trans* (each one of the alleles presenting a different mutation). However, as the child manifests the typical TRPS I heterozygous patients' phenotype, they are probably in *cis*, behaving as a single mutation. In this case, we cannot be certain that both mutations are pathogenic—perhaps one is benign. Additionally, gonadal mosaicism in either of the parents could not be excluded.

A single case of homozygosity for mutated TRPS1 gene has been described in the literature [4]. Ludecke found only a mutant (2681TrA), but no normal TRPS1 allele in one individual from an affected family. The patient presented nearly absent hair and several skeletal anomalies, resembling severe TRPS III. His mother, heterozygous for the missense mutation, manifested TRPS I phenotype. 

### 3.2. Trichologic Abnormalities

Trichologic abnormalities, a key clinical feature of TRPS, range from almost normal hair to severe hypotrichosis, in which case the scalp may be completely bald [5, 12, 17]. In many patients, scalp hair grows slower than in healthy individuals and remains relatively short. Sparseness of hairs may also affect the lateral eyebrows, eyelashes, beard, axillary, and pubic hair [12]. Histologic examination of scalp biopsies, performed by Seitz et al., showed that the impression of sparse hair is mainly caused by thinning of individual hairs, not by an important reduction of hair follicles [12].

Thin, fragile, and slowly growing nails, as seen in our patient, have also been described [18]. Additional nail changes in TRPS I include longitudinal striation, racket nails' appearance, leukonychia, and koilonychia [12].

### 3.3. Skeletal Abnormalities

Skeletal abnormalities in TRPS I vary considerably within families and even between monozygotic twins [4]. The most characteristic radiologic abnormality involves the phalanges (mainly the middle ones) and consists of an enlarged, irregular metaphyseal ending, with the shape of a cone or inverted V, and a thin and deformed complementary epiphysis, which usually leads to premature fusion of the growth plate [4, 19]. Other tubular bones, such as the metacarpals, can be similarly affected [19–21]. Patient's hands and feet may look short and stubby, with clinodactyly or variable angulation (ulnar, radial, or to both sides, at different levels) of at least one finger or toe. 

Cone-shaped epiphyses (CSEs) are often not detectable before 2 years of age [4], although mild metaphyseal concavity—a manifestation of future CSEs—can sometimes be seen during the first year of life [21]. Cone-shaped epiphyses are a frequent finding in other skeletal dysplasias and can also occur in otherwise normal children [19, 20], in this case affecting predominantly the distal phalanx of the thumb and the middle phalanx of the small finger [19]. At 4 years of age, our patient presented with metaphyseal concavity in several phalanges of fingers and toes. 

Epiphyseal changes in TRPS result in postnatal, progressive growth retardation [4]. Skeletal age usually lags behind chronological age until puberty and then accelerates, although final adult height typically rests below the 50th percentile [3, 4, 12]. Growth hormone deficiency and other endocrine disturbances have been described, but their association with the underlying TRPS remains uncertain [12].

Hip malformations, such as *coxa plana*, *coxa magna*, or *coxa vara*, develop in more than 70% of patients [4]. In children, TRPS may mimic Perthes disease [5]; in older patients, hip abnormalities frequently resemble degenerative arthrosis. Other skeletal abnormalities, such as severe osteoporosis [11] and supernumerary teeth [17], have been described. Regular observation of these patients, including bone and joint examination, is of the utmost importance.

### 3.4. TRPS and Attention Deficit/Hyperactivity Disorder

Patients with TRPS I and a submicroscopic deletion or a TRPS1 point mutation usually have normal intelligence [3, 4, 7]. When treated for ADHD, our patient presented normal cognitive function. We have not found in the literature any report of clinical association between TRPS type I and neurobehavioral disorders. Being a common disturbance in childhood, this was probably a coincidence. 

### 3.5. Final Comments

Clinical examination is still the most important tool for diagnosing patients with TRPS. Given the widely variable manifestations, many cases of TRPS I probably remain undiagnosed until a more severely affected family member presents with the classic phenotype.

Although there is currently no curative treatment for this condition, timely orthopedic procedures may correct functional disability or chronic arthralgias. Plastic surgery can also be provided, for severe dysmorphisms or esthetic reasons. 

Mutation analysis allows identification of carriers and subsequent genetic counseling. The molecular study of our patient adds two more to the list of mutations found in TRPS patients.
